# ﻿*Tuberstira pennis* (Coleoptera, Tenebrionidae, Lagriinae), a bizarre new genus and species from South China

**DOI:** 10.3897/zookeys.1251.160506

**Published:** 2025-09-15

**Authors:** Yong Zhou, Jie Yan, Bin Chen

**Affiliations:** 1 Institute of Entomology and Molecular Biology, College of Life Sciences, Chongqing Normal University, Chongqing 401331, China Chongqing Normal University Chongqing China; 2 Chongqing Normal University, Chongqing 401331, China Chongqing Normal University Chongqing China

**Keywords:** Lagriini, long-jointed beetle, morphology, new species, Oriental Region, Statirina, taxonomy, type species

## Abstract

*Tuberstira pennis***gen. et sp. nov.**, a new genus and species within the tribe Lagriini, subtribe Statirina, is described from Guangdong, Guangxi, Yunnan and Hainan, China. The genus is readily distinguished from all known Lagriini genera by: large eyes, nearly contiguous on the ventral surface; centrally elevated pronotal disc; corrugated elytra, with setigerous tubercles in intervals; coarsely punctate femora; and transversely expanded aedeagal apex in lateral view. The habitus, male genitalia, female genital tube, ovipositor and key diagnostic features are illustrated.

## ﻿Introduction

The tribe Lagriini Latreille, 1825 is distinguished from other Lagriinae by the following characters: paired defensive glands situated between abdominal sternites VII and VIII; pronotal lateral margins absent or weakly developed; antennal terminal segments typically elongated (Watt 1974; Aalbu et al. 2023). The tribe has a wide distribution across all biogeographical realms and exhibits particularly high diversity in tropical areas, such as the Afrotropical, Neotropical, and Indo-Malayan realms (Borchmann 1936; Bouchard et al. 2021; Aalbu et al. 2023).

As the most speciose tribe within Lagriinae Latreille, 1825, Lagriini comprises 135 extant genera, accounting for approximately 50% of all Lagriinae genera and 6% of Tenebrionidae Latreille, 1802 (Bouchard et al. 2021; Aalbu et al. 2023). Currently, the tribe is classified into three subtribes: Lagriina Latreille, 1825 with 58 genera, Statirina Blanchard, 1825 with 75 genera, and the more recently reestablished Phobeliina Ardoin, 1961 with two genera (Matthews 1998; Aalbu et al. 2023). The subtribe Statirina can be diagnosed by the following characters: body slender; terminal antennomere elongated; prosternal process narrow or wide, clearly separating procoxae; elytra typically punctate-striate, with subparallel lateral margins (Merkl 1986, 1987; Aalbu et al. 2023).

During our taxonomic research on Chinese Lagriini beetles, five specimens collected from South China bear exceptionally large eyes, a centrally elevated pronotal disc, and elytral intervals with setigerous tubercles. These characteristics distinguish them from all known Lagriini genera and suggest a unique lineage. Based on comprehensive morphological analyses, we herein propose a new genus in Statirina, *Tuberstira* gen. nov., to accommodate these specimens, with *Tuberstira pennis* sp. nov. designated as the type species.

## ﻿Material and methods

Morphological examinations and dissection were performed using a stereomicroscope (Olympus SZ2-ILST). Specimen habitus, diagnostic characteristics, and measurements were captured with a digital stereomicroscope (KEYENCE-VHX-5000). Images were processed, annotated, and assembled into plates using Adobe Photoshop CS6. Quote marks are used to display verbatim label data, while square brackets indicate author interpretations.

The acronyms of institutions where specimens are deposited are as follows:

**CNU**Chongqing Normal University, Chongqing, China;

**MYNU** Invertebrate Collection of Mianyang Normal University, Mianyang, China;

**SNUC** Insect Collection of Shanghai Normal University, Shanghai, China.

## ﻿Results

### ﻿ Tuberstira gen. nov.

Taxon classificationAnimaliaColeopteraTenebrionidae

D60F0686-DA98-5A92-BEC7-0CB2AE1DB9B4

https://zoobank.org/42469ED4-77D8-448B-BD23-EBD937CAAEDA

Figs 1, 2

#### Type species.

*Tuberstira pennis* sp. nov., by present designation.

#### Diagnosis.

**Figure 1.**
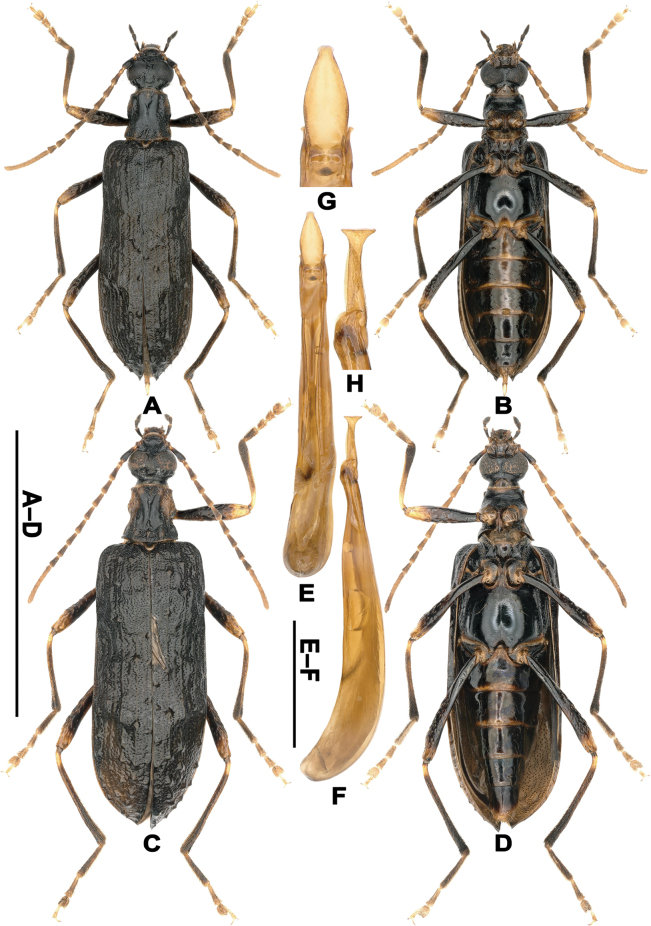
Habitus and aedeagus of *T. pennis* gen. et sp. nov. A, B. Holotype: A. Dorsal view; B. Ventral view; C, D. Paratype (female, Yunnan, Lvchun County, Daxing Town, Bachishan Mountain): C. Dorsal view; D. Ventral view; E, F. Aedeagus: E. Ventral view; F. Lateral view; G, H. Magnified apical portion of aedeagus (not to scale): G. Ventral view; H. Lateral view. Scale bars: 1 cm (A–D); 1 mm (E, F).

**Figure 2.**
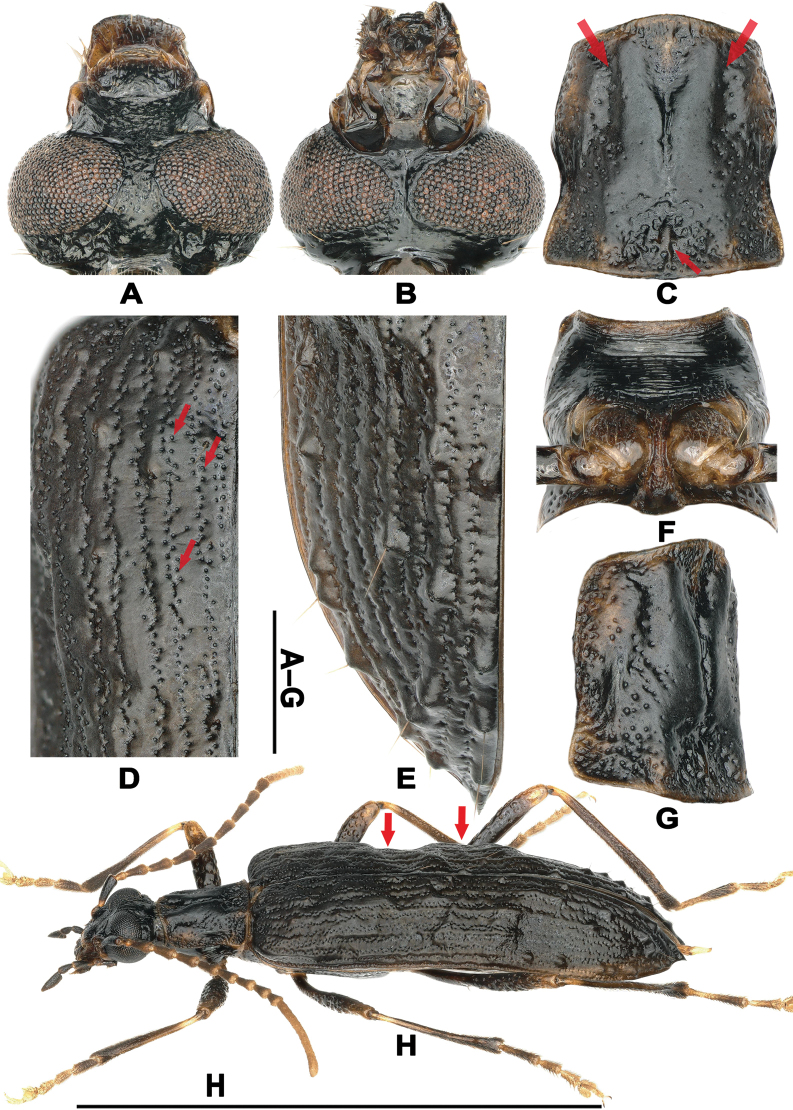
Diagnostic features of *T. pennis* gen. et sp. nov. A, B. Male head: A. Dorsal view; B. Ventral view; C, F, G. Male pronotum: C. Dorsal view (upper arrows indicating longitudinal impressions in lateral portions, the lower indicating the middle carina in posterior 1/5); F. Ventral view; G. Lateral view; D, E. Male left elytron: D. Anterior half (arrows indicating short strial rows in intervals); E. Posterior 1/3; H. The holotype in lateral view (arrows indicating transverse impressions in anterior 3/7 and 4/7, respectively). Scale bars: 1 mm (A–G); 1 cm (H).

Eyes remarkably large, nearly contiguous ventrally (Fig. 2A, B); pronotum with central disc distinctively elevated (Fig. 2C, G); elytra corrugated, with large setigerous tubercles in intervals (Fig. 2E, H); femora with sparse coarse punctures (Fig. 2H); parameres with distinctly transversely expanded apical portion in lateral view (Fig. 1F, H). The new genus exhibits slight resemblance to *Casnonidea* Fairmaire, 1882 and *Sora* Walker, 1859 (same subtribe), but differs from both genera by: head round, with mouthparts moderately protruding forward, mandibles weak (head elongate, with mouthparts strongly protruding forward, mandibles stout in *Casnonidea* and *Sora*); pronotum with central disc elevated (pronotum with central disc not elevated in *Casnonidea* and *Sora*); elytra intervals with setigerous tubercles (elytral intervals impunctate or with few setigerous punctures); femora with sparse coarse punctures (femora impunctate or with sparse minute punctures in *Casnonidea* and *Sora*) (Borchmann 1936; Zhou and Chen 2024). Similar elytral tubercles occur in other Lagriini genera, such as *Kaindilagria* Merkl, 1988 and *Tomogria* Merkl, 1988, but these taxa belong to the subtribe Lagriina (Merkl 1988).

#### Description.

**Male** (Figs 1A, B, 2H). Body elongate, dorsal surface nearly glabrous. Head round. Mouthparts moderately protruding forward; terminal maxillary palpus triangularly elongate; mandibles weak, bending inward, embracing labrum; labrum usually cordiform; labro-epistomal membrane exposed; epistome elevated, higher than labrum. Frons flattened between eyes, with anterior portions gently elevated; frontal canthus moderately swelling. Eyes large, bulging, with anterior margin slightly invaded by frontal canthus, nearly contiguous ventrally. Antennae usually filiform, reaching metacoxae when directed backward, antennomere XI lengthened.

Prothorax widest at base, subequal to head, constricted before base. Pronotum uneven and punctate, with disc elevated centrally; anterior angles obtuse, posterior angles acute; anterior margin moderately arched backward, posterior margin slightly arched forward, both with carina distinctively elevated, lateral portions roundly bending toward ventral surface with the margins invisible in dorsal view. Prosternal process narrow and elevated between coxae, but not as high as coxae, broadened past coxae, and roundly triangular posteriorly (Fig. 2F).

Scutellar shield linguiform, impunctate. Elytra corrugated, with subparallel lateral margins; surface uneven, punctate-striate, strial rows with punctures not contiguous; intervals with setigerous tubercles; humeral callosity not prominent, with dense punctures, rounded in dorsal view, separated from disc by deep impression; elytral margins visible in dorsal view except for the portions beneath humeral callosity; epipleura impunctate, narrow, gradually narrowing toward apex. Metaventrite emarginate apically, convex, higher than metacoxae.

Legs slender; femora slightly clavate, more or less flattened, with sparse coarse punctures; metatarsomere I longest, about as long as metatarsomeres II–IV combined. Abdominal ventrites almost impunctate; ventrite 6 visible.

**Female** (Fig. 1C, D). Body wider. The ratio of interocular distance and eye diameter larger, antennomere XI shorter, the ratio of antennomere XI and the combined length of four preceding antennomeres smaller; the ratio of prothorax length and width smaller; the ratio of elytral length and width smaller.

#### Etymology.

The generic name is a combination of the Latin word “*tuber*” and the customary suffix “-*stira*” for Statirina genera, referring to the prominent setigerous tubercles in the elytral intervals. The name is feminine in gender.

#### Distribution.

China: Guangdong, Guangxi, Yunnan, Hainan.

### ﻿ Tuberstira pennis sp. nov.

Taxon classificationAnimaliaColeopteraTenebrionidae

F79B0D67-F166-5355-BADE-ED9A583390F0

https://zoobank.org/5F402683-D878-4D11-97BD-E95934AB4366

Figs 1, 2, 3, 4

#### Type material

(2 ♂ 3 ♀). ***Holotype***: China • ♂ (Fig. 1A, B); “云南绿春县大兴镇巴尺山” [Yunnan, Lvchun County, Daxing Town, Bachishan Mountain]; 22°57'26"N, 102°26'11"E; alt. 1840 m; 2018.II.20 [adult reared from larva; this date is the larva collection]; “许浩&邱见玥” [Hao Xu & Jian-Yue Qiu leg.]; CNU. ***Paratypes***: China • 1 ♂ [the metatarsomeres I abnormal: the left one notched in outer margin, the right one with a tooth at basal 1/4 of outer margin]; same data as for the holotype; MYNU • 1 ♀ (Fig. 1C, D); same data as for the holotype • 1 ♀; “韶关乳源南岭国家森林公园” [Guangdong, Shaoguan City, Ruyuan County, Nanling National Forest Park]; 24°55'1"N, 113°2'8"E; 2023.V.09; “刘振华” [Zhen-Hua Liu leg.]; night seeking; CNU • 1 ♀; “广西环江县九万山清水塘” [Guangxi, Huanjiang County, Jiuwan Mountain, Qingshuitang]; 25°11'59"N, 108°47'46"E; alt. 450 m; 2021.IV.24; Tang, Peng, Cai & Song leg.; SNUC.

#### Description.

Holotype ♂ (Figs 1A, B, 2H). Body length 12.2 mm, width 3.6 mm. Body elongate, slightly shiny, about 3.39× as long as wide; dorsal surface black, except for lighter labrum, labro-epistomal membrane, anterior and posterior margins, lateral portions of pronotum, scutellar shield; antennae light brown, except for apical 2/3 antennomere I black, antennomere II–X darker; legs black, except for apical portion of femur, basal portions of tibiae, coxae, tarsi light brown; ventral surface black, except for anterior portion of mesepimeron and ventrite 1, posterior margins of metepisternum, abdominal ventrites light brown. Dorsal surface with few long setae scattered on labrum, epistome, tempora and apical portion of elytra; anterior, posterior margins of pronotum, tibiae and tarsi with short setae; ventral surface with sparse long setae.

Head widest at eye level. Terminal maxillary palpus triangularly elongate with straight, cavate inner surface, broadest at base; labrum transversely cordiform, widest before apical margin, slightly emarginate anteriorly; labro-epistomal membrane trapezoidal, widest at base; epistome transversely rectangular with anterior margin slightly arched backward medially, with sparse minute punctures. Frons separated from epistome by indistinct fronto-epistomal impression, with dense, shallow punctures before and between eyes, convex medially with dense, coarse punctures behind eyes (Fig. 2A). Interocular distance about 0.27×, 0.12× as long as eye diameter dorsally and ventrally, respectively (Fig. 2A, B). Antennae filiform, length ratios of antennomeres I–XI as 70: 24: 46: 54: 53: 55: 49: 47: 44: 36: 171, antennomere XI curved with pointed apex, slightly shorter than the combined length of four preceding antennomeres.

Prothorax about 1.08× as long as wide, with dense transverse wrinkles on ventral surface (Fig. 2F). Pronotum sparsely punctate, impressed in lateral portions of apical half; central disc elevated longitudinally, triangularly impressed, punctate before posterior margin and after anterior margin, forming an X-shaped ridge, nearly impunctate in elevated portions, with distinct middle groove, carina in mid 1/3 and posterior 1/5 respectively (Fig. 2C, G); anterior, posterior angles slightly projecting laterally.

Elytra with acute apex (Fig. 2E), 2.46× as long as wide and 4.68× as long as prothorax, transversally impressed in anterior 3/7 and 4/7 (Fig. 2H); strial rows not straight, with punctures separated by distances 1 to 2× puncture diameter; intervals uneven, some with short strial rows adjacent to long strial rows and scattered punctures (Fig. 2D), odd-numbered intervals with large setigerous tubercles, denser in apical 1/3 (Fig. 2E).

Legs thin; profemora slightly flattened in basal 1/3, meso- and metatibiae moderately flattened in basal 1/2; metatibiae flattened, with inner margin slightly curved in posterior 1/2. Abdominal ventrites glossy, with sparse fine setigerous punctures in disc. Defensive glands present, with paired reservoirs attached to the distal 2/3 of ventrite V, the terminal oval-shaped, extending to the basal 2/3 ventrite IV. Aedeagus curved in basal 1/3 in lateral view (Fig. 1F); epinotal pieces of basal piece extending dorsad with acute apex (Fig. 1H); parameres gradually contracted on two sides toward apex, cavate ventrally (Fig. 1E, G), with apex distinctively, transversely expanded in lateral view (Fig. 1F, H).

**Female** (Fig. 1C, D). Body length 14.5 mm, width 4.8 mm. Frons broadly, distinctively separated from epistome by arched forward fronto-epistomal impression, interocular distance about 0.32×, 0.17× as long as eye diameter dorsally and ventrally, respectively, length ratios of antennomeres I–XI as 79: 23: 53: 57: 58: 58: 57: 56: 52: 44: 158, antennomere XI slightly longer than the combined length of three preceding antennomeres; prothorax about 1.02× as long as wide, slightly wider than head, anterior angles acute; elytral length 2.38× as long as width.

**Figure 3.**
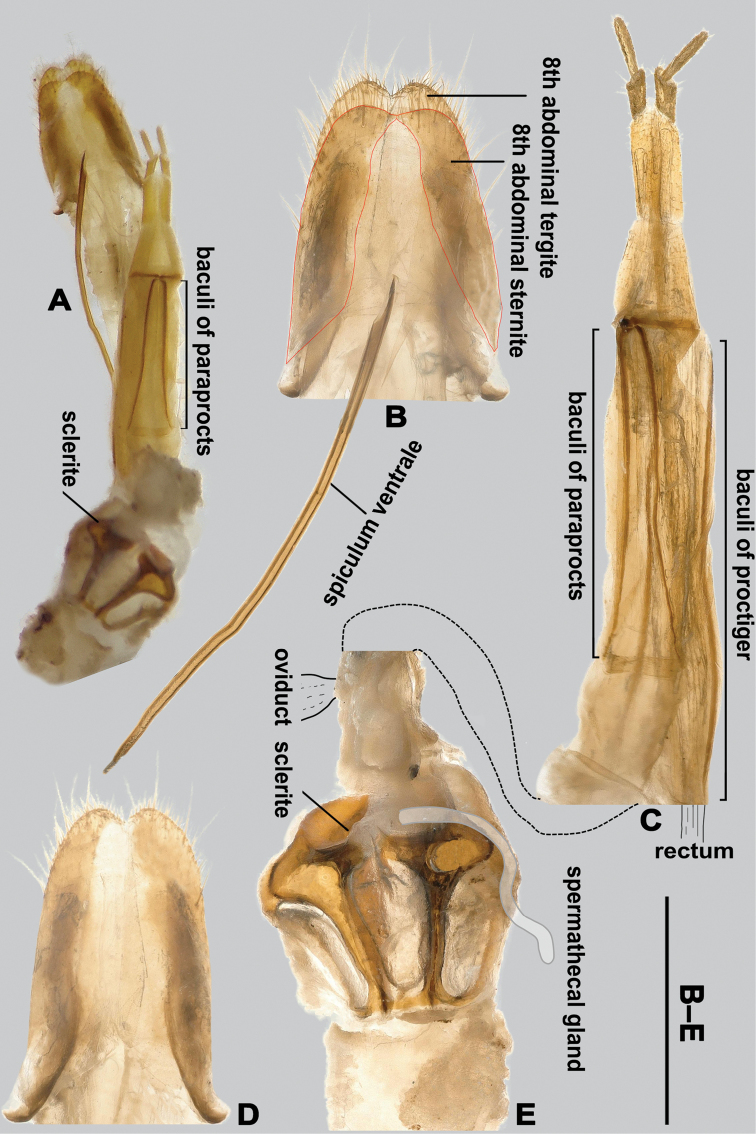
Female 8^th^ abdominal sternite, 8^th^ abdominal tergite, genital tube and ovipositor of *T. pennis* gen. et sp. nov. A. Panoramic photo (not to scale); B. 8^th^ abdominal sternite (highlighted in red outline) and spiculum ventrale; C. Ovipositor; D. 8^th^ abdominal tergite; E. Primary bursa copulatrix, with hand-drawn spermathecal gland and oviduct. Scale bar: 1 mm (B–E).

Spiculum ventrale slender, fused distally (Fig. 3B). 8^th^ abdominal sternite elongate, medially divided into two elongate sclerites (Fig. 3B, D).

Ovipositor (Fig. 3A, C) with paraprocts elongate, 1.5× as long as coxites, baculi of paraprocts longitudinal; 1^st^ coxite lobe short, with baculi transverse; gonostyli terminal; proctiger nearly 1.5× as long as paraprocts, distal end of proctigeral baculi situated far before base of coxites.

Female genital tube with a blind, large primary bursa copulatrix, oviduct and a short spermathecal gland, bursa copulatrix with a large sclerite (Fig. 3A, E).

#### Measurement.

Males: body length 13.4–13.6 mm, body width 3.9–4.0 mm (*N* = 2); females: body length 14.7–15.0 mm, body width 4.8–5.0 mm (*N* = 3).

#### Variability.

Some specimens exhibit entirely brown elytra. In certain individuals, the anterior portions of pronotum are elevated, and the area between eyes is impunctate.

#### Etymology.

The specific epithet is derived from the Latin noun “*pennis*” (meaning “elytron”), referring to the setigerous tubercles on the elytra, in combination with the generic name; noun in apposition.

#### Distribution.

**Figure 4.**
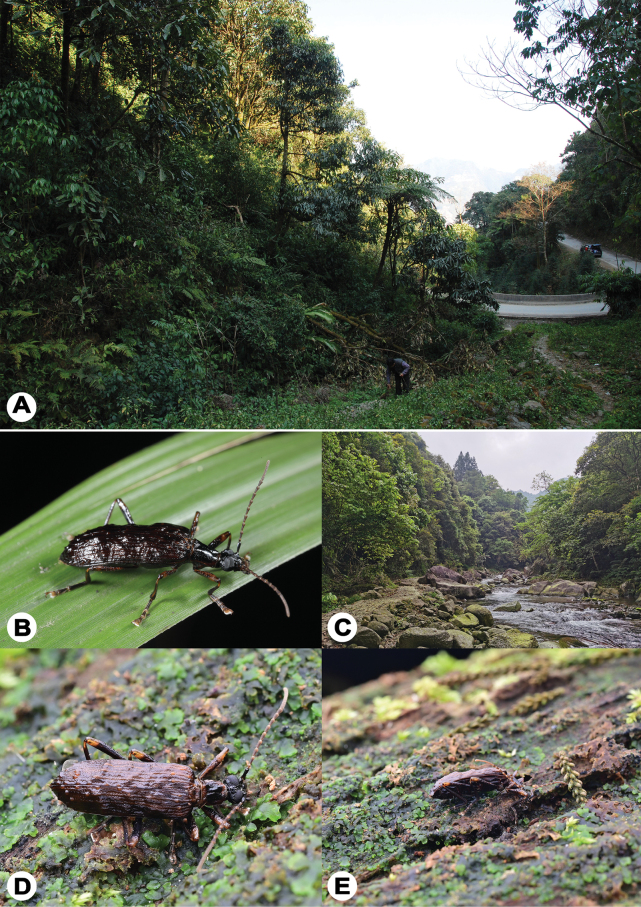
Natural habitats of *T. pennis* gen. et sp. nov. A. Field environment where larvae of three specimens were collected (Yunnan, Lvchun County, Daxing Town, Bachishan Mountain); B. Female resting on grass (Guangdong, Shaoguan City, Ruyuan County, Nanling National Forest Park); C. General habitat of the female specimen (Guangxi, Huanjiang County, Jiuwan Mountain, Qingshuitang); D, E. Male wandering on a decaying log (Hainan, Diaoluo Mountain, near Diaoluoshenshu, 2025.III.15).

China: Guangdong, Guangxi, Yunnan, Hainan (distribution data based solely on photographic evidence, Fig. 4D, E).

#### Ecology.

The three specimens from Yunnan were obtained by rearing larvae excavated from decaying logs (Fig. 4A). The female specimen from Guangdong was collected at night on grass (Fig. 4B). The female specimen from Guangxi was collected by shaking shrubs (Fig. 4C).

## ﻿Discussion

Due to the relatively broad prosternal process (which clearly separates procoxae), a slender body, and punctate-striate elytra, *Tuberstira* gen. nov. is classified within the subtribe Statirina (Merkl 1986, 1987; Aalbu et al. 2023). Although we compared *Tuberstira* gen. nov. morphologically with *Casnonidea* and *Sora* for identification purposes, the latter two genera differ significantly from the new genus (see the “Diagnosis” section of *Tuberstira* gen. nov.). The phylogenetic relationships of the new genus and species within the subtribe still require further study.

Currently, *Tuberstira pennis* gen. et sp. nov. has been recorded in Guangdong, Guangxi, Yunnan and Hainan, with only five specimens collected to date. The records from Guangdong and Hainan were found during night sampling. It is unclear whether its large compound eyes and dark body coloration are directly related to its nocturnal activity. Given that some species in Statirina exhibit distinct flower-visiting behaviors (Zhou et al. 2024), it remains to be seen whether its nocturnal habits are associated with nocturnal pollination. Therefore, resolving these questions will require extensive fieldwork to uncover the life history of this peculiarly shaped species.

## Supplementary Material

XML Treatment for Tuberstira

XML Treatment for Tuberstira pennis

## ﻿Additional information

### Use of AI

No use of AI was reported.

### Author ORCIDs

Yong Zhou https://orcid.org/0000-0002-1411-7563

Jie Yan https://orcid.org/0000-0001-8031-7451

Bin Chen https://orcid.org/0000-0002-5227-7736

### Data availability

All of the data that support the findings of this study are available in the main text.
